# The Lifespan and Turnover of Microglia in the Human Brain

**DOI:** 10.1016/j.celrep.2017.07.004

**Published:** 2017-07-25

**Authors:** Pedro Réu, Azadeh Khosravi, Samuel Bernard, Jeff E. Mold, Mehran Salehpour, Kanar Alkass, Shira Perl, John Tisdale, Göran Possnert, Henrik Druid, Jonas Frisén

**Affiliations:** 1 Department of Cell and Molecular Biology, Karolinska Institute, SE-171 77 Stockholm, Sweden; 2 Center for Neuroscience and Cell Biology, University of Coimbra, 3004-517 Coimbra, Portugal; 3 Institut Camille Jordan, CNRS UMR 5208, University of Lyon, 69622 Villeurbanne, France; 4 Department of Physics and Astronomy, Ion Physics, Uppsala University, 751 20 Uppsala, Sweden; 5 Department of Forensic Medicine, Karolinska Institutet, 171 77 Stockholm, Sweden; 6 NHLBI, NIH, Bethesda, MD 20892, USA

**Keywords:** microglia, human, turnover, proliferation, renewal

## Abstract

The hematopoietic system seeds the CNS with microglial progenitor cells during the fetal period, but the subsequent cell generation dynamics and maintenance of this population have been poorly understood. We report that microglia, unlike most other hematopoietic lineages, renew slowly at a median rate of 28% per year, and some microglia last for more than two decades. Furthermore, we find no evidence for the existence of a substantial population of quiescent long-lived cells, meaning that the microglia population in the human brain is sustained by continuous slow turnover throughout adult life.

## Highlights

• Human microglia renew at a median rate of 28% per year
• Microglial cells are on average 4.2 years old
• Most of the microglia population (>96%) is renewed throughout life

## Graphical Abstract

**Figure:**
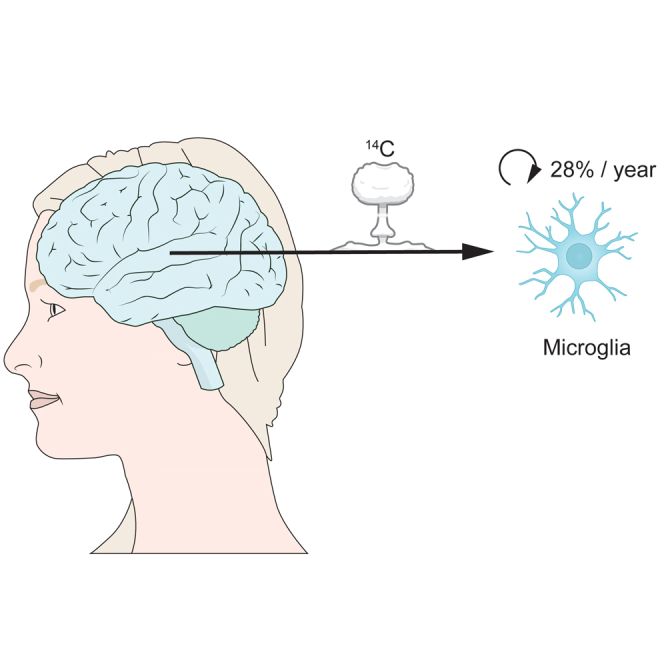

## Teaser

Taking advantage of the decreasing level of atmospheric ^14^C since the Cold War, Réu et al. show that human microglia, unlike most other hematopoietic lineages, slowly turn over at a yearly median rate of 28%. The absence of a large quiescent subpopulation indicates that most microglia will renew throughout life.

## Introduction

Microglia are the resident macrophages of the CNS, which dynamically survey their surrounding for signs of infection or cell distress (Casano and Peri, 2015). In mice, microglial progenitor cells derive from an early myeloid branch of the hematopoietic lineage in the embryonic yolk sac and enter the CNS before the blood-brain barrier is formed (Ginhoux et al., 2010, Schulz et al., 2012). There is no further contribution from the peripheral hematopoietic system under physiological conditions, and this is a self-sustaining population within the CNS (Ajami et al., 2007, Askew et al., 2017, Bruttger et al., 2015, Hoeffel et al., 2015, Mildner et al., 2007). The self-contained nature of this population makes it vulnerable to local disturbances. Additionally, it is key for brain homeostasis that microglial cell numbers are stably maintained, because having reduced numbers results in behavioral and learning deficits (Parkhurst et al., 2013).

Based on [^3^H]thymidine incorporation, heavy water (^2^H_2_O) labeling, and 5-ethynyl-2′-deoxyuridine (EdU) and 5-bromo-2′-deoxyuridine (BrdU) incorporation, 0.075%–1.04% of microglia in adult mice of different strains and 2.35% of microglia in the young adult macaque were estimated to enter the cell cycle each day (Askew et al., 2017, Lawson et al., 1992, Shankaran et al., 2007, Tay et al., 2017, Tonchev et al., 2003). It is difficult to infer cell turnover dynamics in humans from data in experimental animals, which may have very different requirements or lifespans. Assessing the turnover of human immune cells is notably important because it is particularly hard to deduce renewal rates from laboratory animals, which live in pathogen-free barrier facilities (Beura et al., 2016). A recent study demonstrated that 2% of microglia in the adult human brain are in cell cycle at any given time based on Ki-67 labeling, but the authors noted the limitations of this approach and acknowledge the need for more precise measurements (Askew et al., 2017). It is difficult to estimate cell turnover dynamics based on cell cycle markers, because it rests on assumptions of cell cycle length. Moreover, it is not possible to know whether the cell will proceed through the cell cycle to mitosis or whether the potential progeny will survive.

## Results

**Figure 1:**
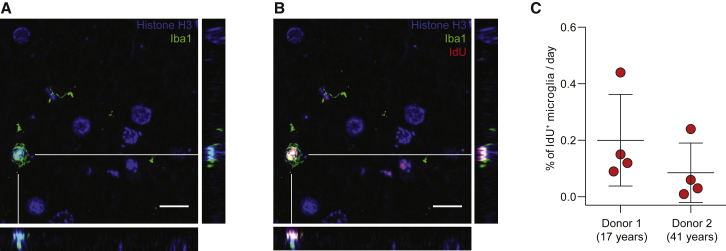
IdU Incorporation (A) Confocal image with orthogonal projections, from human cortex, revealing microglia positive for Iba1. (B) Co-staining of Iba1 and the thymidine analog IdU. (C) Percentage of microglia incorporating IdU per day (mean ± SD). Each data point represents a glass slide. Nuclei are labeled with antibodies to histone H3. Scale bars, 10 μm.

We analyzed the frontal and occipital cortices from two subjects (17 and 41 years old) who had received IdU (5-Iodo-2′-deoxyuridine) as a radiosensitizer for cancer treatment (Table S1). On average, 0.8% of Iba1^+^ parenchymal microglia in the cortex were IdU^+^ after 4 days (donor 1) or 10 days (donor 2) of IdU administration (Figures 1A and 1B; Table S1). Accounting for the labeling period, it averages at 0.14% labeling per day (Figure 1C). These observations do, however, come with the following caveats: (1) the sample size is small and additional samples are not available; and (2) the subjects studied are not healthy individuals, and thus may exhibit aberrant turnover of different populations of cells. Still, our observations provide us with a general estimate of what to expect in our downstream analysis based on retrospective ^14^C measurements.

**Figure 2:**
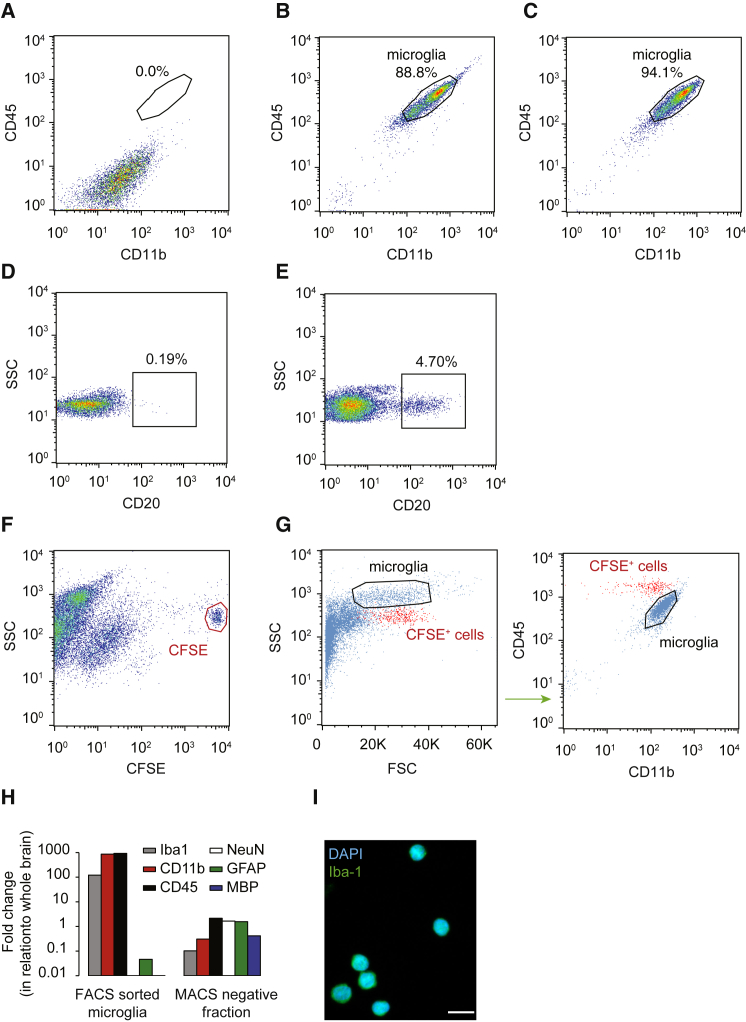
Microglia Isolation (A) Unstained brain single-cell suspension following CD11b isolation with magnetic beads. (B) Sorting gate for FACS sorting of stained microglial cells. (C) Representative post-sort purity of microglia sample. (D) FACS plot representing the percentage of positive cells for the B cell marker CD20 in a brain sample prior to MACS purification. (E) FACS plot representing the percentage of positive cells for CD20 in a sample of peripheral blood. (F) Blood cells were positively selected with CD11b microbeads, labeled with CFSE (red gate), and then spiked into a brain preparation previously selected with CD11b microbeads. (G) Gating strategy for the sorting of microglia (spiked blood cells in red). (H) qRT-PCR reveals that, when compared with the magnetic bead negative fraction, the FACS sorted microglia sample is several hundred-fold enriched for mRNA of the microglia markers Iba1, CD11b, and CD45 and depleted of mRNA for markers of neurons (NeuN), astrocytes (GFAP), and oligodendrocytes (MBP). (I) FACS sorted cells are positive for the microglial marker Iba-1 (A488). Scale bar, 10 μm.

Following CD11b magnetic bead selection, we isolated by fluorescence-activated cell sorting (FACS) CD45^+^/CD11b^+^ microglia from the adult human postmortem cerebral cortex in order to perform retrospective ^14^C dating (Figures 2A–2C) (Olah et al., 2012). We used CD20, a B cell marker, to evaluate the presence of blood-borne cells in dissociated cortical preparations. The percentage of B cells in brain samples prior to MACS purification (Figure 2D) is very low relative to the circulation (Figure 2E), indicating that contamination by blood-borne cells is likely to be a negligible factor. The average postmortem interval of our donors is 43 hr, and it is likely that most blood has coagulated inside the vessels. Nonetheless, we decided to experimentally address the possible contamination with peripheral monocytes, which also express CD45 and CD11b. To do so, we isolated CD11b^+^ cells from peripheral blood with magnetic beads, following the same positive selection protocol we used to isolate microglia from dissociated cortical specimens, and labeled them with carboxyfluorescein succinimidyl ester (CFSE). We then prepared a mixed sample containing both CD11b^+^/CFSE^+^ blood cells and CD11b^+^ microglia isolated in parallel (Figure 2F). This allowed us to simultaneously label and visualize both populations in a single sample, ruling out variability in labeling protocols. Microglia were found to exhibit lower expression of CD45 and did not overlap with blood-borne cells, further suggesting that cells from the blood are not likely to be a significant source of contamination, because cells with higher CD45 expression were excluded in all experiments (Figure 2G). We confirmed the identity of the isolated microglial cells by qPCR using Iba1, CD11b, and CD45 (Tambuyzer et al., 2009), and found only minimal contamination of neurons (NeuN), astrocytes (GFAP), or oligodendrocytes (MBP) (Figure 2H). As expected, the sorted cells are positive for the microglial marker Iba-1 (Figure 2I).

**Figure 3:**
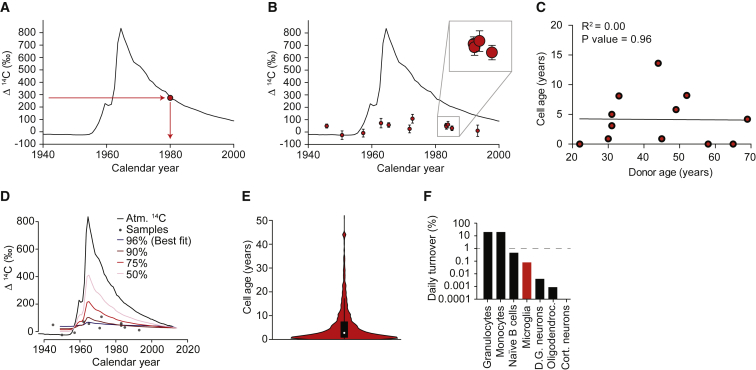
Microglial Population Dynamics (A) Schematic illustration of the ^14^C atmospheric curve over time (Levin et al., 2010). The concentration of ^14^C in the genomic DNA of a cell population is dependent on the atmospheric ^14^C concentration (y axis). Thus, the birth date of the cell population can be read off the x axis. (B) ^14^C content in the genomic DNA of human cortical microglia from donors born across six decades. Data points plotted along the x axis according to the date of birth of the donors. Close-up view of four nearly overlapping data points (gray square). The error bars represent the ^14^C concentration measurement error. (C) Representation of the average age of microglia in each individual and linear regression (black line). (D) Different models for distribution of data on the atmospheric ^14^C curve considering different frequencies of dividing cells. The model that best fits the data are one where most cells (>96%) renew. (E) Considering the turnover rate, the average cell age, and the fact that all microglial cells do not divide simultaneously, we created a stochastic cell age distribution model. Our model shows that within an individual there is a distribution of cells of different ages, with some having recently renewed and others not having divided in more than 20 years (donors with infinite turnover not included). (F) The approximate rate of microglia turnover is 0.08% a day, a low turnover rate in comparison with other immune cells (granulocytes, monocytes, and naive B cells) but a high turnover rate relative to other CNS cells (neurons in the dentate gyrus, oligodendrocytes, and cortical neurons).

By relating the ^14^C level in the DNA of cells to the atmospheric ^14^C curve, one can determine the average date of birth of the cell population (Spalding et al., 2005) (Figure 3A). Using this strategy, we performed retrospective birth dating of microglia isolated from adults born across six decades (Figure 3B; Table S2). Based on the year of collection and date of birth of the sample, it is possible to calculate the average cell age of the sample (Figure 3C). Using a homogeneous turnover model in which each cell is replaced at a fixed rate, it is possible to obtain robust snapshots of cell dynamics for each individual donor (Bernard et al., 2010). In simple terms, this is accomplished by calculating how often cells need to divide within an individual of a given age in order for the cells to have the measured ^14^C level. If cells are as old as the individual, their renewal rate is zero and the measured ^14^C level corresponds to the level at birth. In mice, under homeostatic conditions, microglia have been shown to have a stable network and to self-renew stochastically (Tay et al., 2017). Similarly, in the homogeneous turnover model, all cells are equally likely to be replaced (Supplemental Information). Our results indicate that the majority of microglia in the healthy human cortex are replaced by newly produced cells at a median rate of 28% per year (or 0.08% per day), and that they have an average age of 4.2 years (Figure 3C; Table S2). This is a lower rate than estimated by IdU incorporation (0.14% per day; Figure 1C). The difference may indicate that the microglial population is somewhat heterogeneous because a pulse of IdU preferentially labels a more rapidly dividing subpopulation.

Cells that became postmitotic soon after birth have a unique ^14^C signature, corresponding to the atmospheric level at that moment in time. This allowed us to directly investigate the possible existence of a non-dividing subpopulation, using a heterogeneous turnover model (Figure 3D). The model that best fits our data are one where the majority of the population (>96%) is renewed and we have no evidence to support the existence of a significant subpopulation of quiescent very long-lived cells (Figure 3D). We have employed a conservative mathematical model, with few assumptions, which does not preclude that an extended dataset or other variables could explain the heterogeneity observed.

Based on the average cell age and cell division rate observed, we generated cell age distributions stochastically, showing that within an individual there is a wide range of microglia ages (Figure 3E). Simply because not all cells are dividing at the same time, some are quite young due to recent cell division and others can be more than 20 years old (Figure 3E).

The lower rate of microglia renewal compared with most other immune cells is probably a manifestation of the immune-privileged status of the CNS (Figure 3F) (Busch et al., 2007, Macallan et al., 2005). On the other hand, in comparison with other cells of the CNS, microglia show a high exchange rate (Figure 3F) (Spalding et al., 2005, Spalding et al., 2013, Yeung et al., 2014). Thus, a constant basal renewal is likely necessary for the maintenance of a cohort of young and healthy microglial cells.

## Discussion

Administering nucleotide analogs for a short time period introduces a bias to label the cells with the highest proliferation rate within a potentially heterogeneous population. Also, labeled cells that continue to proliferate after the labeling period will give rise to additional positively labeled cells that lead to overestimations of cell proliferation (Neese et al., 2002). Nevertheless, samples of human brain labeled with nucleotide analogs are very valuable, not only due to their rarity but also as a confirmatory tool of in vivo renewal in humans (Eriksson et al., 1998, Ernst et al., 2014, Yeung et al., 2014).

Analysis of the integration of atmospheric ^14^C, derived from nuclear bomb tests, in genomic DNA is cumulative and gives a more comprehensive view of cell age and cell division history (Spalding et al., 2005), and it is possible to analyze tissue from subjects without previous serious illness. There is little exchange of carbon atoms in genomic DNA in non-dividing cells, and the effect of DNA repair and methylation is well below the detection limit of this retrospective birth dating strategy (Bergmann et al., 2012, Ernst et al., 2014, Spalding et al., 2005), even after, for example, stroke, where there is a substantial increase in DNA damage and repair (Huttner et al., 2014).

Most immune cells do not live longer than a few days or weeks (Busch et al., 2007, Macallan et al., 2005), making microglia one of the slowest dividing immune cells described to date. An extreme exception is plasma cells in the intestine, where the subset with the slowest renewal rate has a median age of 22 years (Landsverk et al., 2017). The turnover rate of microglia in humans is substantially lower than in mice. There is a much higher exchange of oligodendrocytes in mice compared with humans (Yeung et al., 2014), and it is possible that clearance of myelin and cell debris calls for a higher exchange rate of microglia in mice.

Based on Ki-67 staining, a recent study estimated that, at any given moment, 2% of microglia are proliferating in the human brain (Askew et al., 2017). Similarly to short-term labeling experiments using BrdU or IdU, Ki-67 staining detects dividing cells, but it provides limited information for cells that may exhibit slow renewal rates. Importantly, Ki-67 does not measure proliferation directly, and cells blocked in G_1_ or destined to enter apoptosis can accumulate in the population as Ki-67^+^ events because of a G_1_/S block (Busch et al., 2007). An additional explanation for the results of the different techniques resides in the fact that newborn microglia are more likely to die than the resident microglia (Askew et al., 2017). Hence, many of the cells detected by Ki-67 and, to a lesser extent, by IdU may be destined to die and not to replace existing ones.

In conclusion, ^14^C analyses reveal that microglia as a whole turn over slowly, and that individual cells can potentially be decades old. Complementary, IdU and Ki-67 (Askew et al., 2017) measurements suggest heterogeneity within the population possibly induced by the presence of a subpopulation of fast dividing cells. Finally, our data predict a nearly complete renewal of the microglia population in the human cortex during the lifespan of an individual, similar to what has been seen in mice (Askew et al., 2017).

## Experimental Procedures

### Tissue Collection

Neocortical tissue was obtained from donors admitted for autopsy at the Department of Forensic Medicine in Stockholm from 2014 to 2016, after informed consent from the relatives. The ethical permit for this study was granted by the Regional Ethics Committee of Stockholm (2010/313-31/3). Formalin-fixed and paraffin-embedded sections of cortical frontal lobe and occipital lobe from cancer patients, who had received IdU for therapeutic purposes, were obtained from the National Heart, Lung and Blood Institute, NIH. Buffy coats were obtained from anonymous regular blood donors at Blodcentralen, Karolinska University Hospital.

### IdU Quantification

The sections were immersed in xylene to remove the paraffin, and the tissue was rehydrated in descending ethanol series. Triton X-100 (0.2%) was used to permeabilize the tissue, and antigen retrieval was performed in 0.05% citraconic acid solution (pH 7.4) for 20 min in a domestic steamer. The sections were left for 20 min at room temperature and then immersed in 2.0 N HCl for 40 min. The slides were blocked (10% donkey normal serum in PBS with 0.2% Triton X-100) at room temperature for 1 hr. Following incubation with the primary antibodies (1:60 mouse anti-BrdU, BD347580; 1:100 goat anti-Histone H3, Abcam 12079; 1:100 rabbit anti-Iba1, Wako 019-19741), the sections were incubated with the secondary antibodies (1:200 donkey anti-mouse Cy3, Jackson ImmunoResearch 715-165-150; 1:200 donkey anti-goat A647, Jackson ImmunoResearch 705-605-147; 1:200 donkey anti-rabbit A488, Jackson ImmunoResearch 711-545-152) and inspected in an LSM 700 (Carl Zeiss) confocal microscope. The percentage of IdU^+^ microglia in each slide was found based on the total number of Iba^+^ cells and the number of Iba1^+^/IdU^+^ cells. By dividing the percentage of labeled cells by the number of labeling days (4 for donor 1 and 10 for donor 2), we calculated the daily labeling. The raw data and the calculations are in Table S1.

### Tissue Dissociation

After careful removal of the meninges and all visible blood vessels, the tissue was cut into small pieces and thoroughly rinsed with PBS. The tissue was then homogenized in media A (1× HBSS, 150 mM HEPES, 2 mM EDTA, 5% BSA) with 2 U/mL papain (Worthington) and 10 U/mL DNaseI (Roche) at 37°C for 1.5 hr. The homogenized tissue was mix with 3 volumes of sucrose media I (PBS, 0.7 M sucrose, 2 mM EDTA) and centrifuged for 20 min at 1,000 × *g*. The pellet was then resuspended in sucrose media II (PBS, 0.9 M sucrose, 2 mM EDTA) and centrifuged for 25 min at 800 × *g*. Finally the pellet was resuspended in blocking solution (PBS, 0.1% FBS, 2 mM EDTA) and filtered through a 40 μm cell strainer.

### Cell Isolation

The cell suspension was incubated for 5 min with human Fc-gamma receptor (FcR)-binding inhibitor (1:100; eBioscience) and for 30 min with CD11b antibody-conjugated microbeads (1:25, 130-093-634; Miltenyi Biotec). The magnetic isolation was performed according to the manufacturer. The samples were next incubated for 20 min with PE-CD11b (1:20, clone ICRF44; BioLegend) and Alexa 647 CD45 (1:20, clone HI30; BioLegend), and finally FACS sorted in an Influx flow cytometer (BD Biosciences). Blood cells were isolated from buffy coats by density gradient (Lymphoprep). Peripheral blood mononuclear cells (PBMCs) were positively selected with CD11b antibody-conjugated microbeads (1:25, 130-093-634; Miltenyi Biotec) according to the manufacturer.

### DNA Isolation

In order to prevent carbon contaminations, we performed the DNA isolation in a clean room (ISO8). The extraction protocol was modified from Miller et al. (1988). The glassware was prebaked for 4 hr at 450°C. 1 mL of lysis buffer (100 mM Tris [pH 8.0], 200 mM NaCl, 1% SDS, and 5 mM EDTA) and 12 μL of Proteinase K (40 mg/ml) were added to the sorted cells and incubated at 65°C overnight. The samples were further incubated at 65°C for 1 hr after 6 μL of RNase cocktail (Ambion) was added. 600 μL of NaCl (5 M) was added to the sample; then it was vortexed for 30 s. The solution was spun down at 13,000 rpm for 6 min. The supernatant containing the DNA was transferred to a 12 mL glass vial. Ethanol 95% (6 mL) was added and the glass vial was manually agitated. The DNA precipitate was washed three times in DNA washing buffer (70% ethanol [v/v] and 0.5 M NaCl), dried at 65°C overnight, and resuspended in 0.5 mL DNase/RNase free water (GIBCO/Invitrogen). The DNA purity and concentration were verified by UV spectroscopy (NanoDrop).

### Accelerator Mass Spectrometry

DNA samples suspended in 0.5 mL of water were lyophilized to dryness in a vacuum centrifuged at 2,000 rpm for 2 hr. To convert the samples into graphite, we added excess CuO to each dry sample, and the quartz tubes were evacuated and sealed with a high temperature torch. The tubes were placed in a furnace set at 900°C for 3 hr to combust all carbon to CO_2_. The gas was then purified by freezing the residual water at −80°C, as well as cryogenically trapping the CO_2_ at −196°C and discarding all the other gases. The CO_2_ was chemically reduced to graphite in the presence of zinc powder and iron catalyst in individual miniaturized reactors at 550°C for 6 hr. Thorough laboratory protocols are exercised to minimize the introduction of stray carbon into the sample (Salehpour et al., 2013a). Graphite targets were pressed into individual cathodes and are measured at the Department of Physics and Astronomy, Ion Physics, Uppsala University (Salehpour et al., 2013a, Salehpour et al., 2013b, Salehpour et al., 2015) using the 5 MV Pelletron Tandem accelerator. Large CO_2_ samples (>100 μg) may be split, and δ^13^C are measured by stable isotope ratio mass spectrometry, which established the δ^13^C correction to −24.1‰ ± 0.5‰ (1 SD), which was applied to the samples. Corrections for background carbon introduced during sample preparation were made as described previously (Salehpour et al., 2013a, Salehpour et al., 2013b, Salehpour et al., 2015). The measurement error was determined for each sample and ranged between ±8‰ and 40‰ (2 SD) Δ^14^C for the large (>100 mg C) and small samples (10 μg C), respectively. All ^14^C data are reported as Fraction Modern F ^14^C as defined in Reimer et al. (2004) or Δ^14^C as defined in Stuiver and Polach (1977). All accelerator mass spectrometry (AMS) analyses were performed blind to the identity of the sample.

### qRT-PCR

qRT-PCR was performed using TaqMan gene expression assays (RBFOX3, 4331182; AIF-1, 4331182; ITGAM, 4331182; PTPRC, 4331182; GAPDH, 4331182; and MBP, 4331182).

## Author Contributions

P.R. and J.F. designed the study. P.R. performed most of the experiments. A.K. performed the IdU analyses. S.B. did all of the mathematical analyses. J.E.M. performed additional experiments. M.S. and G.P. did the AMS measurements. K.A. and H.D. collected and classified the samples for ^14^C. S.P. and J.T. collected and classified the IdU samples. P.R., J.E.M., and J.F. wrote the manuscript.

## Supplemental Information

Document S1. Supplemental Experimental Procedures and Tables S1 and S2

Document S2. Article plus Supplemental Information
