# Supplementary material for: The Lifespan and Turnover of Microglia in the Human Brain
Source: Cell Rep. 2017 Jul 25;20(4):779–84. doi: 10.1016/j.celrep.2017.07.004 (PMC5540680; doi:10.1016/j.celrep.2017.07.004)
Supplement: Document S1. Supplemental Experimental Procedures and Tables S1 and S2 [file mmc1.pdf]

Cell Reports, Volume *20*

Supplemental Information

The Lifespan and Turnover

of Microglia in the Human Brain

Pedro Réu, Azadeh Khosravi, Samuel Bernard, Jeff E. Mold, Mehran Salehpour, Kanar Alkass, Shira Perl, John Tisdale, Göran Possnert, Henrik Druid, and Jonas Frisén

Supplemental Information - Mathematical modeling

"Bombcurve" age

A straightforward way to estimate the age of a sample is to subtract from the date of collection the calendar year of formation corresponding to the ^{14}C level of the sample. The corresponding "bombcurve" ages are a good estimate of the average age of the sample, when samples are not too old.

The birth-and-death equation

A more precise estimate of the age of a ^{14}C sample is obtained by using birth-and-death models. Birth-and-death models describe the dynamics (the evolution in time) of a cell population in an individual, in which cells are born and die. In an individual aged *t* years, we denote by $N(t)$ the *cell number* in the cell population. The atmospheric ^{14}C data provide information on the birthdates of the cells; it is natural to track the age of the cells. The *chronological age* of a cell is defined here as the time elapsed since its last division. To take into account the age of the cells, we break down the population $N(t)$ into a continuum of age bins. This introduce a population structured in chronological cell age *a*. The new dynamical variable $n(t,a)$ is the *cell density* at age *a* at time *t*. The *cell number* and the *cell density* are related in the following way

$$N(t) = \int_0^\infty n(t,a)\,da\,.$$

The cell density is expressed in *cells per year.* To specify fully the birth-and-death dynamics, we must set the *initial conditions*, the *birth rate* and the *death rate*. Each set of initial conditions, birth rate and death rate defines a model for the evolution of a cell population over the lifetime of an individual.

Homogeneous turnover model

The simplest model is the one where the cell number is fixed, and the birth and death rates are constant (**homogeneous turnover model**). All dying cells are replaced with a newborn cell, and all cells, old and young, are equally likely to be replaced. The cell death rate is the rate at which cells are replaced, and is termed turnover rate.

This model is well suited when the average age of the cells is relatively small (less than ~10 years). The "bombcurve" age estimate confirmed that the microglial cells were relatively young, with an average of 4.2 years. Because young samples are associated to high cell turnover, more complex models that take into account lifelong changes in turnover dynamics cannot be used. The birth-and-death equations for the homogenenous turnover model are

(PDE) $$\underbrace{\frac{\partial n(t,a)}{\partial t}}_{\text{change in person age}} + \underbrace{\frac{\partial n(t,a)}{\partial a}}_{\text{change in cell age}} = \underbrace{-\gamma n(t,a)}_{\text{loss of cells}},$$

(Initial condition) $$\underbrace{n(t=0,a)}_{\text{cells present at birth}} = \underbrace{N_0\delta(a)}_{\text{all cells aged 0 at birth}} \;,$$

(Boundary condition) $$\underbrace{n(t,a=0)}_{\text{newborn cells}} = \underbrace{\gamma N_0}_{\text{cell birth}} \quad \text{for } t \in [0,t].$$

The first equation is a linear partial differential equation (PDE) that is often used in population dynamics (Perthame, 2007). It is a biological transport equation, with the term transport used in the sense that cells are transported along their age at a unit speed (i.e. they get older). The negative sign on the right-hand-side of the PDE indicates cell loss due to death. The initial condition states that cells are initially aged 0. The Dirac delta-function δ takes a value zero when *a* is not 0, and is normalized so that the total cell number

$$\int_0^\infty N_0\delta(a)\,da = N_0.$$

According to the PDE, cells can only die but no source term for new cell is provided. We need to supplement the PDE with a special condition for newborn cells. The boundary condition specifies the birth rate of the cells ($n(t, a = 0)$). The equations are valid on domain $a \in [0,t]$, and $t \in [0,t]$, where *t* is the age of the individual at time of sample collection.

Heterogeneous turnover model

An alternative to the homogeneous model is a model where only a subset of the cells is susceptible to renewal. In that model, the homogeneous model is applied to a fraction *f* of the cell population, while a fraction (1-*f*) is assigned a 14C level corresponding to the year of birth of the donor.

Fitting the birth-and-death equations to the data

Computing the ^{14}C concentration associated to a model

Solutions for the cell density $n(t,a)$ can be expressed explicitly by (Bernard et al., 2010)

$$n(t,a) = N_0\delta(t-a)e^{-\gamma a} + N_0\gamma e^{-\gamma a}.$$

The first term accounts for the cells that were formed during development, and the second term accounts for the cells that were born after development. The total cell number, obtained by integrating n(t,a) with respect to age a, is N0. The average ^{14}C level $\tilde{C}$ of a DNA sample of a person born at calendar year D_{birth} and collected at calendar year D_{coll}, is

$$\tilde{C} = \frac{\int_0^{t=D_{coll}-D_{birth}} \overbrace{K_{lag}(D_{coll}-a)n(t,a)}^{\text{contribution of cells aged } a \text{ to 14C}} \, da}{\underbrace{N(t)}_{\text{total number of cells}}}.$$

The function K_{lag} is a food-lag atmospheric ^{14}C level curve. When evaluated at calendar year y, $K_{\text{lag}}(y)$ represents the actual ^{14}C concentration that will integrate into new DNA, and will therefore correspond to an average of past atmospheric concentration, to account for the food supply chain, from photosynthesis to the table.

Here, we used a discrete shift function: $K_{\text{lag}}(y) = K(y - t_{\text{lag}})$ with $t_{\text{lag}} = 1$ year (Spalding et al., 2013). This means that the atmospheric carbon would take at time t_{lag} to reach dividing cells. ^{14}C content measured from blood serum in Swedish residents revealed a lag of 1.5 ± 0.7 years (Georgiadou et al., 2013), very close to the lag we used.

Individual turnover rates estimates with the homogeneous turnover model

The homogeneous model has a single parameter, the turnover rate γ, which can be estimated for each individual sample by solving the scalar equation $\tilde{C}(\gamma) = C_{measured}$ for γ. The initial cell number N_0 does not enter explicitly in the equation for $\tilde{C}$, since it appears as a factor on the numerator and the denominator.

Global estimates with the heterogeneous turnover model

The heterogeneous turnover model has two parameters, and cannot be fitted to individual donor, because there would not be a unique solution. Rather, the model was fitted to all the samples, and parameters estimated in the least-square sense, where the sum-of-square of the residuals $\sum(\tilde{C} - C_{measured})^2$ is minimized.

Numerical Methods

All simulations were performed with MATLAB (version R2012b). Solutions for the PDEs and the carbon concentration model were integrated numerically. The atmospheric ^{14}C level curve was sampled at mid-point each year (1993.5, 1994.5, …) and linearly interpolated to convert it to a continuous function for use in the numerical integral functions.

Supplemental References

Bernard, S., Frisén, J., and spalding, K.L. (2010). A mathematical model for the interpretation of nuclear bomb test derived 14C incorporation in biological systems. Nucl Instr and Meth *268*, 1295-1298.

Georgiadou, E., Stenstrom, K.E., Uvo, C.B., Nilsson, P., Skog, G., and Mattsson, S. (2013). Bomb-pulse 14C analysis combined with 13C and 15N measurements in blood serum from residents of Malmo, Sweden. Radiation and environmental biophysics *52*, 175-187.

Perthame, B. (2007). Transport equations in biology. Frontiers in Mathematics.

Spalding, K.L., Bergmann, O., Alkass, K., Bernard, S., Salehpour, M., Huttner, H.B., Bostrom, E., Westerlund, I., Vial, C., Buchholz, B.A., *et al.* (2013). Dynamics of hippocampal neurogenesis in adult humans. Cell *153*, 1219-1227.

Table S1 – Included subjects and IdU data, related to figure 1.

Donor	Age (years)	Cortical region	Per slide<br>Iba1+ cells	Per slide<br>Iba1+/IdU + cells	Per slide<br>% of IdU+ microglia	Per slide<br>% of labeled cells / day	Diagnosis	IdU administration (days)
Donor 1	17	frontal	1194	21	1,76	0,44	Metastatic Osteosarcoma. The dura and other meninges are unremarkable. The cerebrum contains 4 metastatic tumor nodules, all of which are superficial and form hard balls easily detached from the underlying cerebral tissue, which is compressed in those areas. From these nodules, one is located in the left frontal lobe measuring 1 x 1 cm, one in the left parietal lobe measuring 1. 5 x 1 cm and two in the occipital lobes bilaterally, measuring: 1 x 2 cm and 1. 5 x 3 cm correspondingly. The deeper cerebral substance and the cerebellum are free of tumor. Microscopy: The tumor nodule in the left parietal lobe appears pleomorphic. All nodules show an enormous amount of osteoid production. The osteoid is focally necrotic probably on a result to the radiotherapy.	4
			3224	15	0,47	0,12		
		occipital	2516	9	0,36	0,09		
			1479	9	0,61	0,15		
Donor 2	41	frontal	2573	3	0,12	0,01	Recurrent atrial fibro sarcoma (high-grade III sarcoma). Rare microscopic areas of recent ischemic infection in the cerebral cortex.	10
			1223	7	0,57	0,06		
		occipital	614	2	0,33	0,03		
			451	11	2,44	0,24		
					0,8 AVERAGE	0,14 AVERAGE		

Table S2 – Included subjects and 14C data, related to figure 3.

Sample ID	Donor age (years)	Donor birth	Year of sample collection	Sex	Infections	Cause of death	Postmortem interval	Immuno labeling	Tissue	Estimated carbon mass according to measured DNA	Carbon mass measured in graphitization reactor
ND306	31	1983/05	2014	M	negative	NA	50,6h	$CD45^+/CD11b^+$	Cortex	8,87	8,54
ND308	69	1945/10	2014	M	negative	Accident (drown)	37h	$CD45^+/CD11b^+$	Cortex	36,80	29,64
ND311	52	1962/11	2014	M	negative	Suicide	25h	$CD45^+/CD11b^+$	Cortex	5,30	5,20
ND312	49	1965/04	2014	F	negative	Suicide (hanging)	51,9h	$CD45^+/CD11b^+$	Cortex	34,78	28,92
ND328	31	1983/06	2014	M	negative	Suicide (hanging)	49,4h	$CD45^+/CD11b^+$	Cortex	16,52	13,85
ND345	30	1985/03	2015	M	negative	Suicide (Intoxication)	37h	$CD45^+/CD11b^+$	Cortex	15,10	11,01
ND351	22	1993/04	2015	F	negative	Accident (Intoxication)	31h	$CD45^+/CD11b^+$	Cortex	2,98	2,77
ND353	58	1957/04	2015	F	negative	Accident	51h	$CD45^+/CD11b^+$	Cortex	5,98	5,50
ND357	65	1950/08	2015	M	negative	Coronary arteriosclerosis	45h	$CD45^+/CD11b^+$	Cortex	5,72	5,44
ND368	45	1971/11	2016	M	negative	Accident (Intoxication)	61h	$CD45^+/CD11b^+$	Cortex	7,73	6,50
ND373	44	1972/10	2016	M	negative	Suicide (hanging)	48h	$CD45^+/CD11b^+$	Cortex	4,03	2,81
ND374	33	1983/12	2016	M	negative	Suicide (hanging)	35h	$CD45^+/CD11b^+$	Cortex	8,40	6,29

Sample ID	Δ 14C (‰)	Δ 14C Error (2SD)	F 14C	F 14C Error (2SD)	NanoDrop A260	NanoDrop A280	NanoDrop 260/280	NanoDrop 260/230	Turnover rate (yearly)	Cell age (years)
ND306	52,28	18,40	1,060	0,018	1,06	0,54	1,96	2,20	0,199	5,0
ND308	48,62	16,20	1,057	0,016	4,42	2,25	1,96	2,26	0,236	4,2
ND311	71,88	38,20	1,080	0,038	0,64	0,34	1,89	2,11	0,122	8,2
ND312	56,91	18,40	1,065	0,018	4,18	2,10	1,99	2,26	0,172	5,8
ND328	43,45	20,20	1,052	0,020	1,98	1,01	1,96	2,22	0,322	3,1
ND345	30,20	18,60	1,038	0,019	1,81	0,90	2,02	2,28	1,056	0,9
ND351	10,41	45,80	1,018	0,046	0,36	0,18	1,94	2,13	Inf	0,0
ND353	-8,43	31,60	0,999	0,032	0,72	0,37	1,92	2,19	Inf	0,0
ND357	-24,93	34,40	0,983	0,034	0,69	0,35	1,97	2,38	Inf	0,0
ND368	25,22	31,67	1,033	0,032	0,93	0,49	1,91	2,29	1,084	0,9
ND373	107,53	35,25	1,116	0,035	0,48	0,26	1,90	2,23	0,070	13,6
ND374	59,81	26,46	1,068	0,026	1,01	0,52	1,93	2,32	0,122	8,1
